# Steric Interference in Bilayer Graphene with Point Dislocations

**DOI:** 10.3390/nano9071012

**Published:** 2019-07-14

**Authors:** Francisco Arca, Juan Pedro Mendez, Michael Ortiz, Pilar Ariza

**Affiliations:** 1 Escuela Técnica Superior de Ingeniería, University of Seville, 41092 Seville, Spain; 2 Division of Engineering and Applied Science, California Institute of Technology, Pasadena, CA 91125, USA

**Keywords:** graphene, force constants model, distributed dislocations

## Abstract

We present evidence of strong steric interference in bilayer graphene containing offset point dislocations. Calculations are carried out with Large-scale Atomic/Molecular Massively Parallel Simulator (LAMMPS) using the Long-Range Carbon Bond-Order Potential (LCBOP) potential of Los et al.. We start by validating the potential in the harmonic response by comparing the predicted phonon dispersion curves to experimental data and other potentials. The requisite force constants are derived by linearization of the potential and are presented in full form. We then continue to validate the potential in applications involving the formation of dislocation dipoles and quadrupoles in monolayer configurations. Finally, we evaluate a number of dislocation quadrupole configurations in monolayer and bilayer graphene and document strong steric interactions due to out-of-plane displacements when the dislocations on the individual layers are sufficiently offset with respect to each other.

## 1. Introduction

Bilayer graphene was reported by Noselov et al. [1], who described graphene configurations containing one, two and three atomic layers. Interest in monolayer and bilayer graphene stems from their outstanding electronic and mechanical properties [2,3,4], including high thermal mobility, above 4000 W/mK [5], high electronic conductivity, above 15,000 cm${}^{2}$/Vs [6], low mass density, 0.77 mg/m${}^{2}$, and high breaking strength [7]. Owing to these exceptional properties, in conjunction with ever improving production techniques, graphene remains a material of interest for potential application in next-generation electronic devices [8,9].

The physical properties of graphene are strongly influenced by the presence of defects within the lattice. For instance, partial dislocations in graphene give rise to profound changes in transport properties [10,11] and interesting topological states [12]. For this reason, graphene defects, such as dislocations, vacancies, grain boundaries or doped graphene, have been studied using different interatomic potentials and several computational approaches, from *ab initio* methods to molecular dynamics. One of the most common graphene and carbon nanotube defect structures is the Stone-Wales [13], which is composed of two pentagon-heptagon cell pairs resulting from the rotation of a single atomic bond through 90${}^{\circ }$. Meyer et al. [14] examined this kind of defect using transmission electron microscopy (TEM). Li et al. [15] calculated the formation energy and the activation barrier of the Stone-Wales defect using the density-functional theory. Xiao et al. [16] studied the deterioration of the mechanical properties of graphene due to the presence of this type of defects by using an atomistic based finite bond element model. It is found that the Stone-Wales defect modifies the chemical [17] and electronic [18,19] properties of pristine graphene lattices. 7-5 pair structures can also combine to form dislocation dipoles [20]. Lehtinen et al. [21] have applied high-energy electron irradiation to graphene lattices triggering vacancy-type defects, observing that for a certain number of vacancies the atoms locally reorganize into a dipole terminated by two edge dislocations. Warner et al. [22] have explained that dislocation dipoles can also be formed during the CVD growth, through the addition of surface adatoms or to Stone-Wales bond rotations. Jeong et al. [23] studied the stability of dislocation dipoles using density-functional theory.

Carbon structures have been analyzed using a number of interatomic potentials ranging from harmonic potentials expressed in terms of force constants [24,25] to nonlinear potentials [26,27], more accurate but computationally costlier. The latter category includes reactive potentials, e.g., the Reactive Empirical Bond-Order (REBO) potential introduced by Brenner [28]. The addition of torsion and non-bonded interactions to this potential further resulted in the AIREBO potential [29]. Subsequently, Los et al. [30] developed the Long-Range Carbon Bond-Order Potential (LCBOP) and its second version, the LCBOPII potential [31], which accounts for interatomic interactions up to fourth neighbors. This latter potential is similar to AIREBO but presumed to be better suited to large displacements, extreme pressures and temperatures, liquid-solid phases and crystal lattices with topological defects.

In this paper, we present evidence of strong steric interference in bi-layer graphene containing offset point dislocations resulting from the out-of-plane displacements of the individual layers. Calculations are carried on the Large-scale Atomic/Molecular Massively Parallel Simulator (LAMMPS) [32] of Sandia National Laboratories using the LCBOP [30,31] interatomic potential. We start by presenting an assessment of the fidelity of the LCBOP potential [30,31], cf. Section 2, in applications to graphene. In Section 2.1, we start by validating its harmonic response by comparing the predicted phonon dispersion curves to experimental data of Siebentritt et al. [33], Oshima et al. [34], Nicklow et al. [35] and Yanagisawa et al. [36] and those of other potentials. The requisite force constants are derived by linearization of the potential and are presented in full form in [37]. Subsequently, we turn to the anharmonic behavior of the LCBOP potential. Specifically, in Section 2.2 we evaluate the potential in configurations involving the formation of dislocation dipoles and quadrupoles in monolayer graphene and assess its ability to predict accurate and physically meaningful structures. With the LCBOP potential thus validated, we turn to the characterization of the core structure and energies of dislocation quadrupoles in bilayer graphene. Remarkably, we find that, if the dislocations in the individual layers are sufficiently offset, the out-of-plane displacements of the layers give rise to strong steric interactions which result in high energies. A summary of the main conclusions is presented in Section 4 by way of closure.

## 2. Validation of the Theoretical Model

For completeness and subsequent reference, we begin with a brief summary of the LCBOP potential [30,31]. The potential energy $E_{b}$ of a system of *N* atoms is given by

(1) $$E_{b}=\frac{1}{2}\sum_{i,j}^{N}\left(S_{sr,ij}^{\mathrm{down}}V_{ij}^{\mathrm{sr}}+S_{sr,ij}^{\mathrm{up}}V_{ij}^{\mathrm{lr}}+\frac{1}{Z_{i}^{mr}}S_{mr,ij}^{\mathrm{up}}V_{ij}^{mr}\right),$$

where the short-range term $V_{ij}^{\mathrm{sr}}$ gives the energy of the covalent bonds and $V_{ij}^{\mathrm{lr}}=V^{\mathrm{lr}}\left(r_{ij}\right)$ describes long-range interactions, with $r_{ij}=\left|r_{i}-r_{j}\right|$ the distance between atoms *i* and *j*. $V_{ij}^{mr}$ is a term for bond breaking and formation based on *ab initio* calculations of the dissociation energy curves for single, double and triple bonds. It vanishes if the material is in a single phase, as for graphene. The *S* functions are switches expressed in terms of the Heaviside step function, Θ. $V^{\mathrm{sr}}$ is a Brenner type bond-order potential defined as

(2) $$V_{ij}^{\mathrm{sr}}=V_{R,ij}^{\mathrm{sr}}-B_{ij}V_{A,ij}^{\mathrm{sr}},$$

where $V_{R,ij}^{\mathrm{sr}}$ and $V_{A,ij}^{\mathrm{sr}}$ are repulsive and attractive radial pair potentials,

(3) $$V_{R,ij}^{\mathrm{sr}}=A^{\mathrm{sr}}e^{-\alpha r_{i}}$$

and

(4) $$V_{A,ij}^{\mathrm{sr}}=B_{1}^{\mathrm{sr}}e^{-\beta_{1}r_{i}}+B_{2}^{\mathrm{sr}}e^{-\beta_{2}r_{i}},$$

respectively. The bond-order term $B_{ij}$ takes into account several many-body phenomena. It takes the form

(5) $$\begin{array}{c} B_{ij}=\frac{1}{2}\left(b_{ij}+b_{ji}\right)+F_{ij}^{\mathrm{conj}}+T_{ij}, \end{array}$$

where $b_{ij}$ is a bond angle between first neighbors, $F_{ij}$ describes conjugate systems and $T_{ij}$ takes torsion into account. Long-range interactions are accounted for through the pair potential

(6) $$V^{\mathrm{lr}}=\left[\Theta \left(r_{0}-r\right)V_{1}^{\mathrm{lr}}\left(r\right)+\Theta \left(r-r_{0}\right)V_{2}^{\mathrm{lr}}\left(r\right)\right]S_{\mathrm{lr}}^{\mathrm{down}}\left(r\right),$$

where $V_{i}^{\mathrm{lr}}\left(r\right)\left(i=1,2\right)$ are Morse functions,

(7) $$V_{i}^{\mathrm{lr}}\left(r\right)=\epsilon_{i}\left(e^{-2\lambda_{i}\left(r-r_{0}\right)}-2e^{-\lambda_{i}\left(r-r_{0}\right)}\right)+v_{i}.$$

We note that all these functions are differentiable up to their first order and continuous up to their second order. Detailed expressions of the first and second derivatives of the potential up to fourth neighbors may be found in [37]. These derivatives are subsequently used in the calculation of force constants for the 18 neighbors of a reference atom.

### 2.1. Harmonic Response

**Figure 1:**
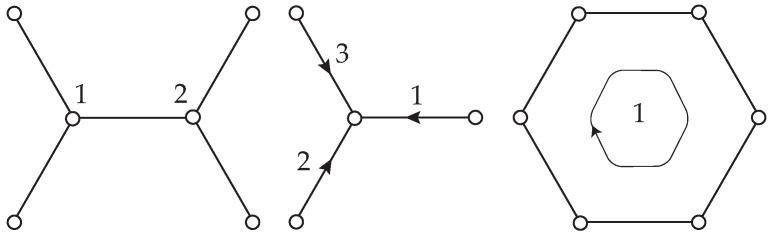
0-cells (atoms), 1-cells (atomic bonds) and 2-cells (hexagonal areas) in graphene.

We begin by assessing the LCBOP potential as applied to graphene in the harmonic range. To this, we consider an infinite and perfect graphene lattice consisting of atoms, bonds and hexagonal cells [38], cf. Figure 1. We note that there are two types of atoms in the lattice, according to the collection of bonds connected to the atoms. Following standard notation [39], we label the atoms in the lattice with the double index (l,α), where $l=(l^{1},l^{2},l^{3})$ are integer lattice coordinates and α=1,2 designates the type of atom. The lattice force constants are, then,

(8) Φl−mαβ=∂2Etot∂r(l,α)∂r(m,β),

where $E^{\mathrm{tot}}$ is the total energy of the lattice and rlα are the spatial coordinates of atom (l,α). The force constants depend on l−m only due to translation invariance. A systematic application of invariance under the symmetry group of graphene shows that the matrices Φl−mαβ are necessarily of the form

(9) $$\Phi_{i}=-{\left(\begin{array}{ccc} \alpha_{i} & 0 & 0\\ 0 & \beta_{i} & 0\\ 0 & 0 & \delta_{i} \end{array}\right)}_{i=1,3},$$

(10) $$\Phi_{2}=-\left(\begin{array}{ccc} \alpha_{2} & \gamma_{2} & 0\\ -\gamma_{2} & \beta_{2} & 0\\ 0 & 0 & \delta_{2} \end{array}\right),$$

(11) $$\Phi_{4}=-\left(\begin{array}{ccc} \alpha_{4} & \gamma_{4} & 0\\ \tau_{4} & \beta_{4} & 0\\ 0 & 0 & \delta_{4} \end{array}\right),$$

where the subscripts represent the set of neighbors. Finally, an application of the discrete Fourier Transform furnishes the representation

(12) Φijl−mαβ=1(2π)2∫−ππ∫−ππΦ^ijθαβe−iθ·(l−m)dθ1dθ2,

where the functions Φ^ijθαβ characterize the phonon dispersion curves of the material [39].

**Figure 2:**
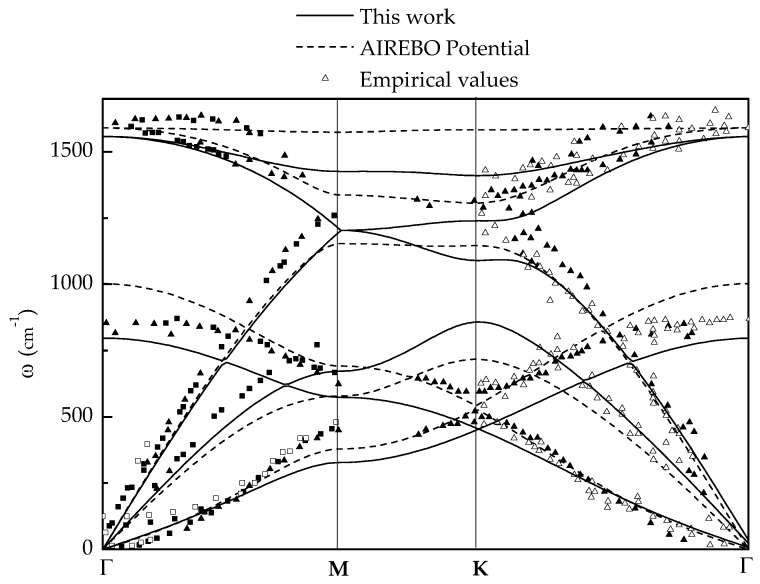
Comparison between the experimental point values of phonon dispersion obtained by Siebentritt et al. [33] (empty triangles), Oshima et al. [34] (empty squares), Nicklow et al. [35] (filled squares) and Yanagisawa et al. [36] (filled triangles); the phonon dispersion curves calculated using the AIREBO potential [40] and those calculated in the present work.

Table 1 shows a comparison between the LCBOP constants, as computed from the expressions given in [37], and those put forth by Wirtz and Rubio [25], Tewary and Yang [27], Ariza et al. [38,40] and Mendez et al. [18]. As can be seen from the table, there are significant differences between the various potentials even in the harmonic range. Figure 2 compares the phonon dispersion curves predicted by the LCBOP and the AIREBO potentials and the experimental data of Siebentritt et al. [33], Oshima et al. [34], Nicklow et al. [35] and Yanagisawa et al. [36]. As can be seen from the figure, the fidelity of the LCBOP and AIREBO phonon dispersion curves is comparable.

### 2.2. Lattice Defects

We proceed to assess the fidelity of the LCBOP potential in a number of configurations including dipoles and quadrupoles in monolayer configurations.

#### 2.2.1. Method of Analysis

**Figure 3:**
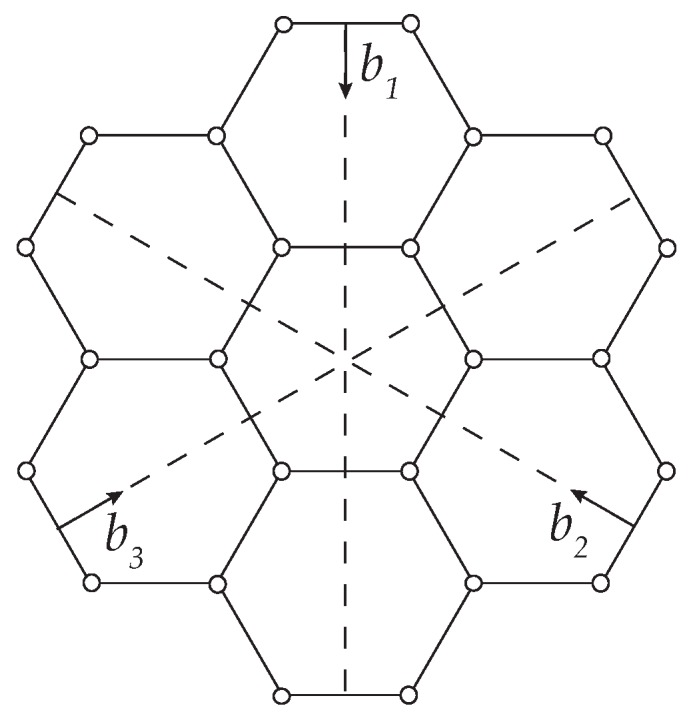
Burgers vectors of graphene.

**Figure 4:**
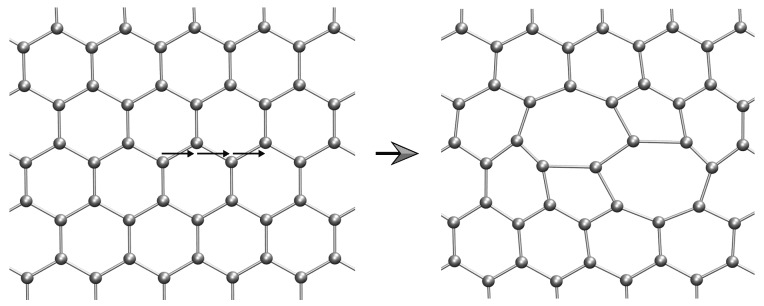
Unrelaxed Stone-Wales defect configuration generated by gliding along three consecutive zig-zag bonds.

We introduce discrete dislocations in graphene by means of Mura’s theory of eigendeformations [41], as developed by Ariza et al. [42,43]. Specifically, the dislocations are introduced by slip on the three effective slip systems in graphene shown in Figure 3. Stable dipolar configurations are obtained when the unit slips occur across a chain of zig-zag bonds [43]. For an example, gliding along three consecutive zig-zag bonds leads to a 7-5-5-7 or Stone-Wales defect, cf. Figure 4.

**Figure 5:**
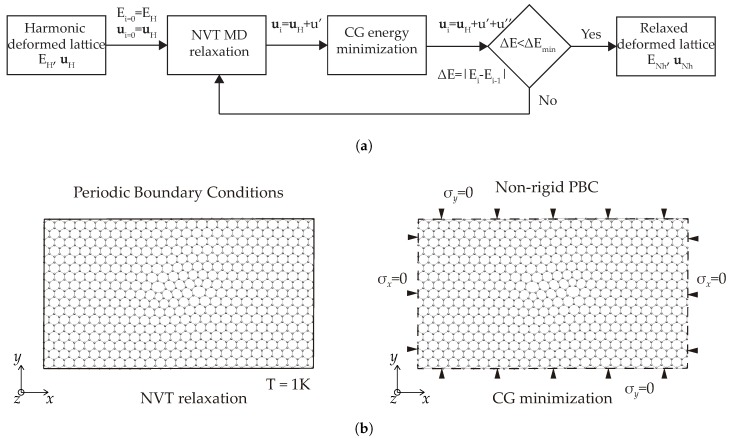
(**a**) Flowchart of the nonlinear relaxation scheme and (**b**) outline of the boundary conditions for the nonlinear relaxation steps.

We seed calculations by solving for the equilibrium configuration of a prescribed distribution of lattice defects in the harmonic range using the force constants introduced in Section 2.1. Conveniently, such solutions can be obtained analytically in closed form in the Fourier domain. Next, in order to elucidate the fully-relaxed configurations of the defect, we use the harmonic atomic positions as initial conditions for a LAMMPS [32] calculation using the LCBOP [30]. For each defect configuration, we conduct two relaxation steps, as shown schematically in the flowchart in Figure 5. The first step consists of a molecular dynamics NVT relaxation at temperature T=1 K and imposing periodic boundary conditions. In the second step, a conjugate-gradient molecular-statics iteration is performed, giving the equilibrium configuration of the defects. In all cases, we have carried out studies in order to ensure convergence with respect to cell-size. For the harmonic calculations cell-size convergence is attained for 3360 atoms, while the anharmonic calculations require 18,720 atoms.

#### 2.2.2. Dislocation Dipoles

As a first validation case, we study a periodic dislocation dipole configuration of increasing length and compute the corresponding harmonic and fully-relaxed equilibrium configurations and energies. We use the number *n* of gliding atomic bonds to measure the size of the dipoles. The linear size *d*, measured from the center of the rotating bonds in the undeformed lattice, follows as $d=\sqrt{3}a\left(n-1\right)/2$, where a=1.42Å is the interatomic distance.

**Figure 6:**
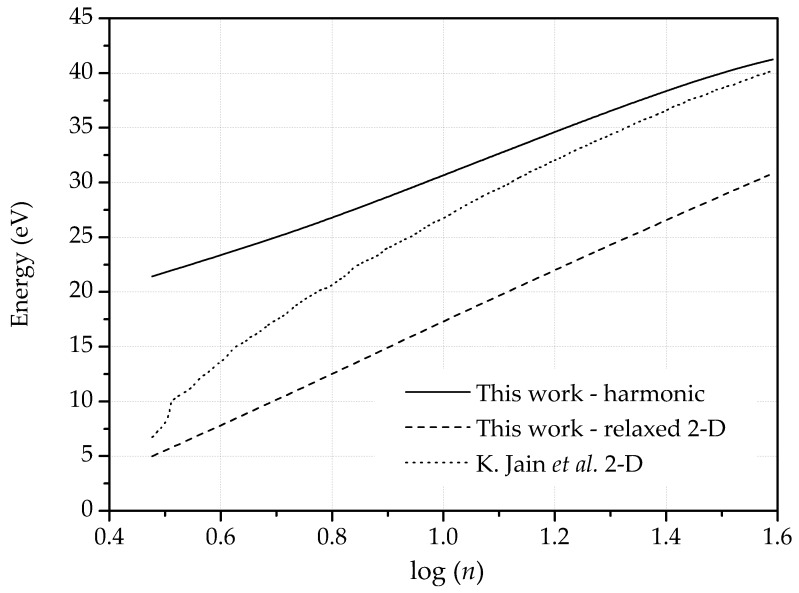
Dislocation energy as a function of dipole size, *n*, in 2-D with the force constants model, after relaxing harmonic configurations and presented by S.K. Jain et al. [44].

We start by constraining the out-of-plane displacements. Under these conditions, the energy exhibits the expected logarithmic dependence with dipole size, Figure 6, in accordance with previous studies [38]. As expected, the anharmonic energies are much lower than the harmonic ones. Thus, the energy of the SW defect, or shortest dipole, decreases from 21.4 eV to 5.0 eV when the configuration is fully relaxed. This energy value is in keeping with—but lower than—the values 5.96 eV and 5.92 eV reported in [44,45]. It is also noteworthy that in the presence of defects the LCBOP is considerably softer than the semiempirical potential of Jain et al., which accounts for second-neighbor interactions only.

**Figure 7:**
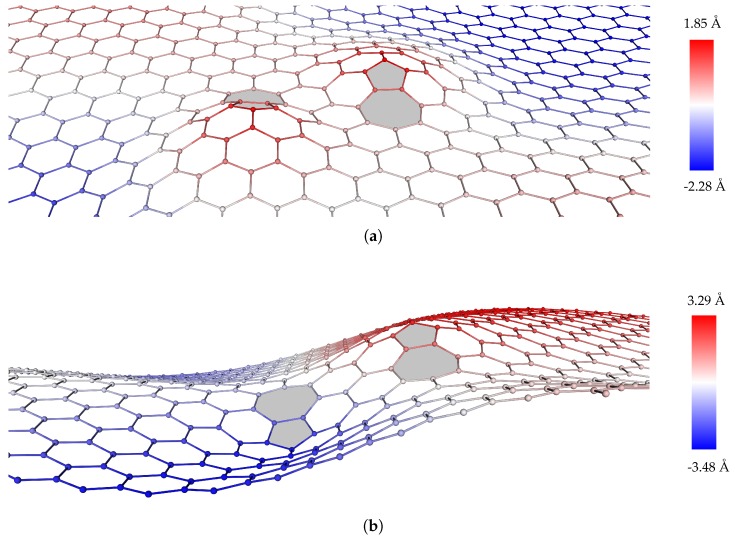
Symmetric (**a**) and antisymmetric (**b**) stability modes in graphene. The color code indicates the out-of-plane position of the atoms.

Remarkably, the fully relaxed 3-D dipole configurations exhibit two different stable modes: a symmetric, or *S*, mode in which the dislocation cores move in the same out-of-the plane direction; and an antisymmetric, or *AS*, mode in which the dislocation cores move in opposite out-of-plane directions, cf. [21], Figure 7. Since the stored energy per dislocation core of the *S* mode is approximately 10% higher than the corresponding *AS* mode, we regard the *S* mode as metastable, cf. [44]. In calculations, we prime the *AS* mode—and eschew the *S* mode—by applying an initial small out-of-plane displacement of the order of 0.2 Å to one atom in each core in opposite directions to each other. For the Stone-Wales defect, we compute a out-of-plane displacement range ($\Delta z=z_{\max}-z_{\min}$) of 2.6 Å, comparable to the value of 2 Å reported by [46].

**Figure 8:**
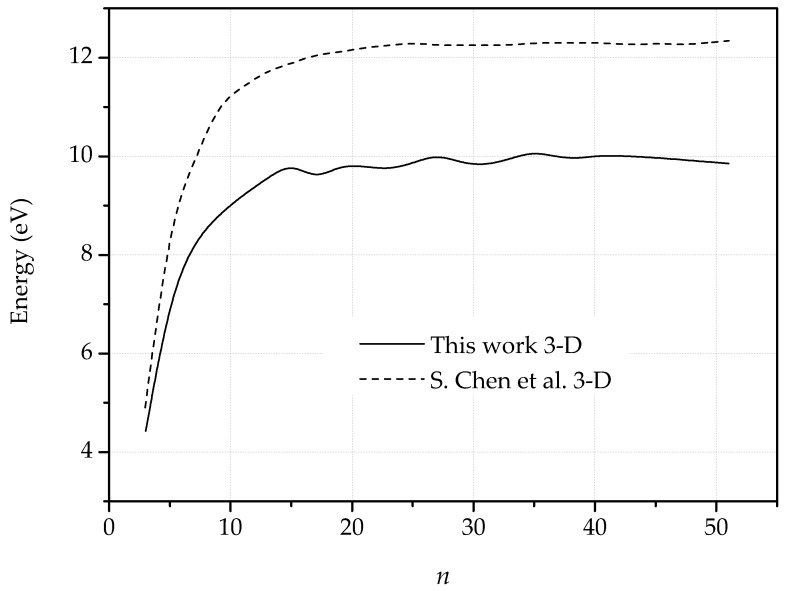
Dislocation energy as a function of the size of the dipole for the 3-D fully relaxed configurations compared with the results presented by Chen et al. [47].

When out-of-plane displacements are allowed, the energy dependence on dipole size is also remarkably different than in the planar case, Figure 8. Thus, following an initial increase up to n=13 approximately, the energy per unit periodic cell attains a constant value ostensibly independent of the dipole size. In particular, the logarithmic dependence characteristic of the planar solutions is lost. Correspondingly, the dislocation cores constrict when out-of-plane dislocations are allowed. The out-of-plane displacements thus effective localize the elastic field of the dislocations, which behave as non-interacting standing solitons. The results of Chen et al. [47] based on the REBO potential show similar behavior, although their maximum stored energy value is ∼2.5 eV higher, Figure 8. This comparison shows that in the presence of defects the LCBOP is considerably softer than the REBO potential.

#### 2.2.3. Dislocation Quadrupole

**Figure 9:**
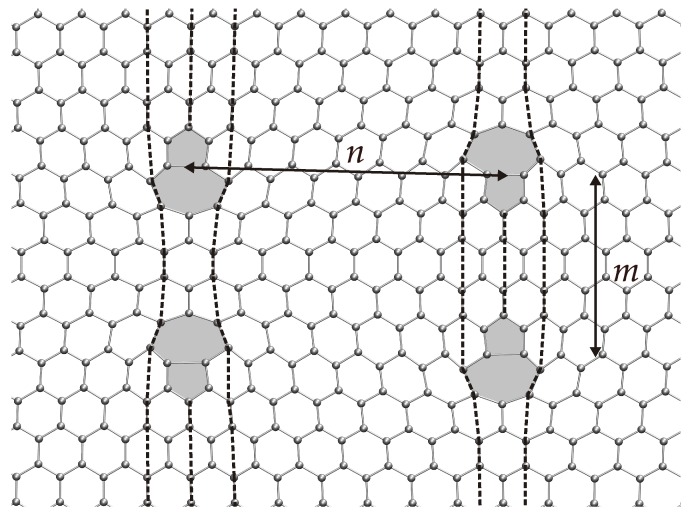
Deformed configurations of periodic quadrupolar arrangement of discrete dislocations for the harmonic solution exhibiting pentagon– heptagon core structures (n=15,m=11).

**Figure 10:**
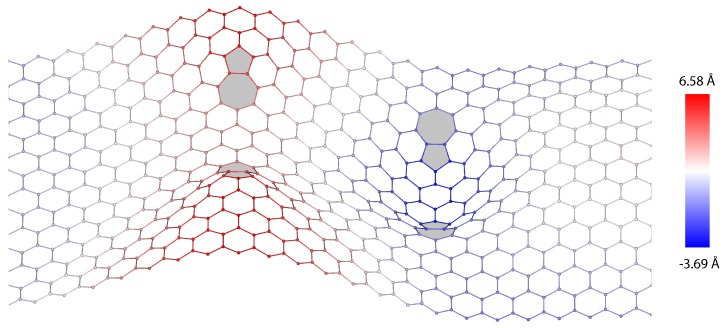
More stable mode of deformed configurations of periodic quadrupolar arrangement of dislocations in graphene.

Dislocation quadrupolar arrangements are composed of two parallel dislocations of equal length and opposite Burgers vector, and thus encompassing four dislocation cores (Figure 9). Here, the periodic configurations are characterized by two parameters: the dipole length *n* and the dipole separation *m*, measured as the number of arm-chair bonds between the dipoles. The lowest energy state is always achieved when the out-of-plane displacements of the dislocation cores are the antisymmetric mode shown in Figure 10, corresponding to the periodic cell shown in Figure 9.

**Figure 11:**
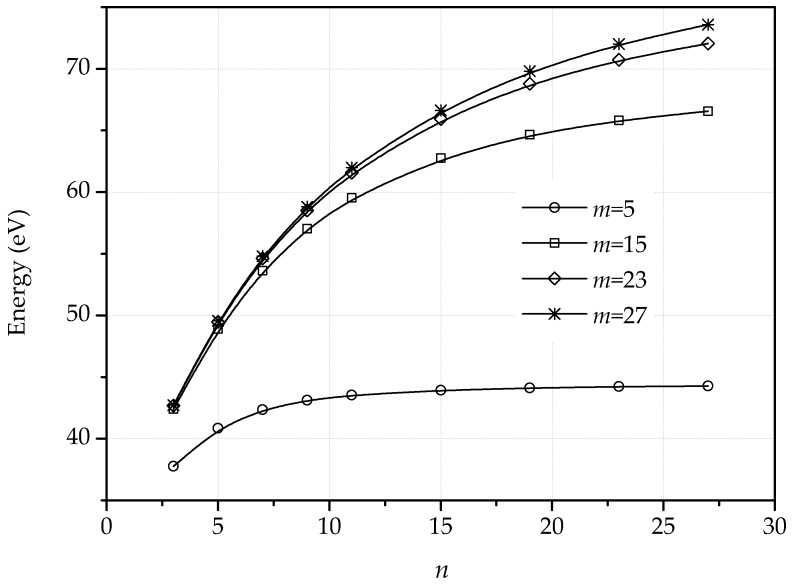
Stored energy of periodic dislocation quadrupoles as a function of dipole sizes, *n*, for different separation between dislocation lines, *m*, using the harmonic model.

**Figure 12:**
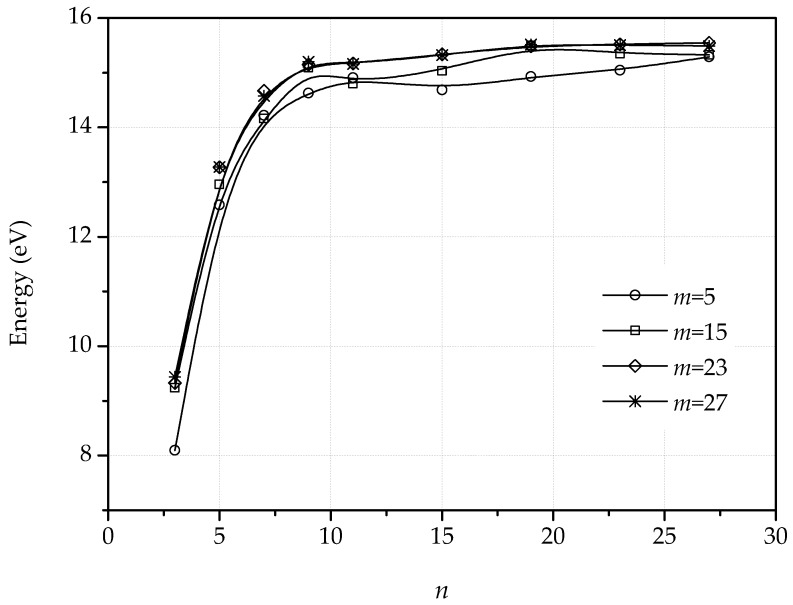
Stored energy of dislocation quadrupoles for the nonharmonic relaxation scheme as a function of dislocation lengths, *n*, for different separations between dislocation lines, *m*.

Proceeding as in the dipole calculations, we have computed the harmonic and nonharmonic atomic positions and stored energy per unit periodic cell of several quadrupolar configurations. We specifically aim to ascertain how the energy depends on the geometric of the quadrupoles, i.e., on *n* and *m*. The planar energies, Figure 11, exhibit the expected logarithmic or dipole-dipole elastic interactions, depending on geometry. Figure 12 shows the energy per unit periodic cell of the fully relaxed quadrupolar configurations. As in the case of dipoles, the out-of-plane displacements confer the dislocations a standing soliton character, with the result that the dislocation interactions are lost for sufficiently large quadrupoles.

## 3. Dislocations in Bilayer Graphene

**Figure 13:**
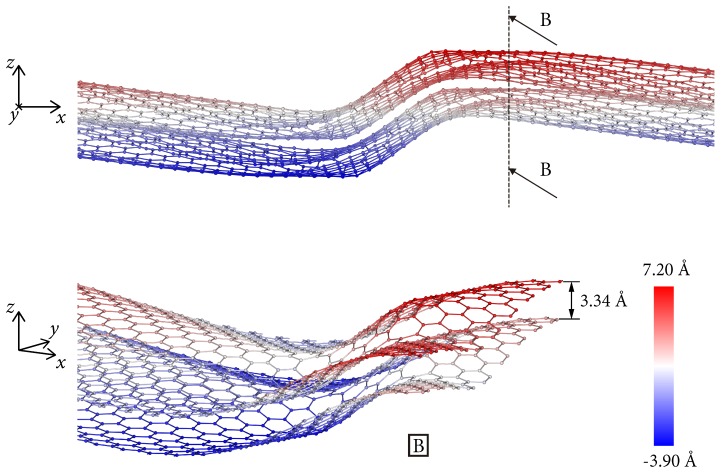
Front (**top**) and diagonal view B (**bottom**) of a bilayer arrangement with one n=9 dipole in each layer, both at the same position in the xy plane. The color code indicates the out of plane position of atoms.

In bilayer graphene, the out-of-plane displacements induced by dislocations can give rise to complex steric interactions between the layers. If the dislocations in both layers are in registry, steric interactions are minimized and the energy of the bilayer is approximately double the energy of one monolayer. An example is shown in Figure 13, corresponding to two n=9 dipoles. The energy of the dipoles in a single layer is 7.96 eV, whereas the bilayer energy is 15.70 eV, or almost double the monolayer energy. The deficit between twice the monolayer energy and the bilayer energy is indicative of a modest attractive interaction between the layers.

**Figure 14:**
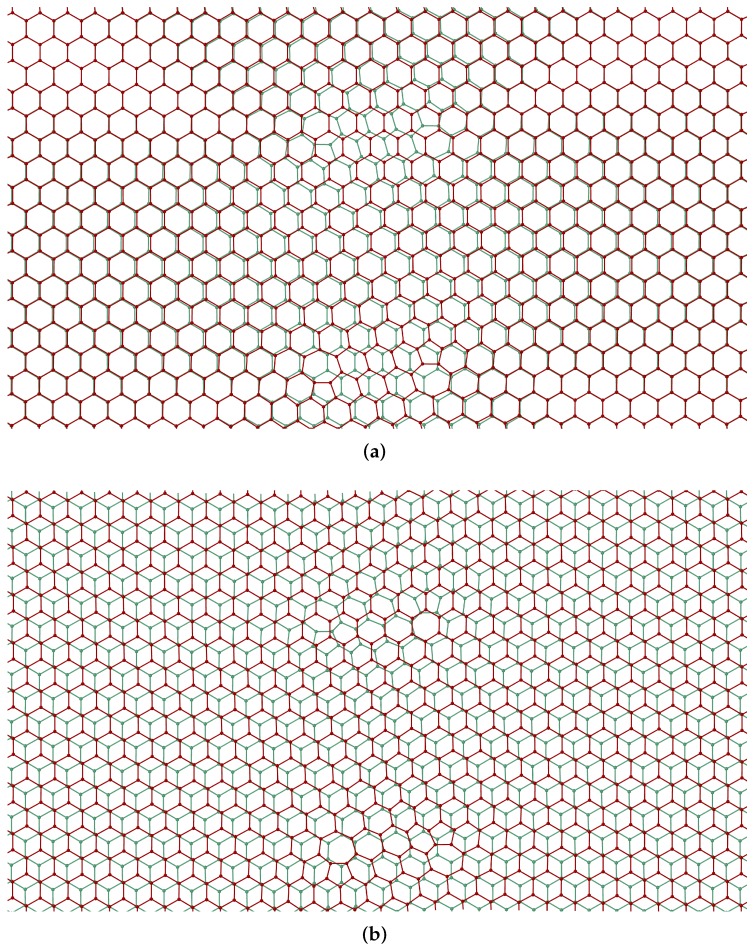
(**a**) Initial AA stacking of bilayer graphene with unmatched dipoles. (**b**) Relaxed configuration showing dislocation core structure and AB stacking.

The preferred stacking of the layers is illustrated in Figure 14. The figure shows the relaxation of a bilayer containing two unmatched dislocation dipoles. In the initial condition, the stacking sequence of the bilayer is AA, Figure 14a. Upon relaxation, the bilayer effects a transition into an AB stacking sequence, Figure 14b, which is the known ground state of bilayer graphene [48].

By contrast, the introduction of unmatched defects in the layers results in strong steric interactions as the layers deflect out of their planes. For example, in a configuration with a n=3 dipole in the bottom layer and a n=11 dipole in the top layer, with the center of both dipoles at the same planar location, the energy of the bilayer defect is 15.3 eV, whereas the sum of the energies of the monolayer defects individually is 3.3 eV lower. Increasing the size of the top dipole to n=21 further increases the energy difference to 4.0 eV. This effect also occurs when only one layer contains a defect. For instance, the n=15 dipolar configuration in a monolayer has a stored energy of 8.5 eV, whereas the same defect in one of the layers of a bilayer has an energy of 13.7 eV.

**Figure 15:**
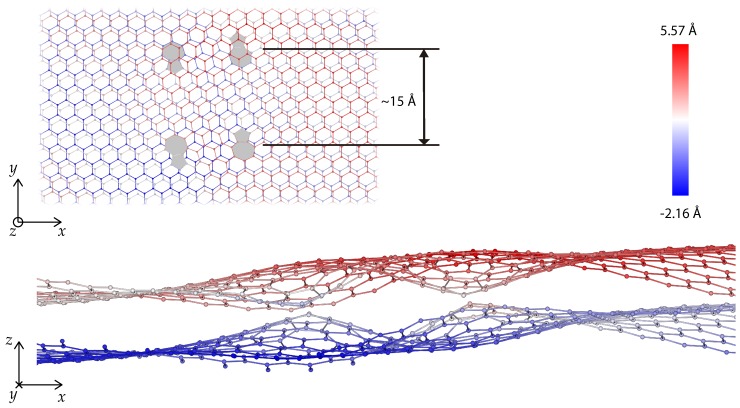
Top (**top**) and front (**bottom**) view of a bilayer arrangement with one n=9 dipole in each layer, separated 15 Å. The color code indicates the out of plane position of atoms.

**Figure 16:**
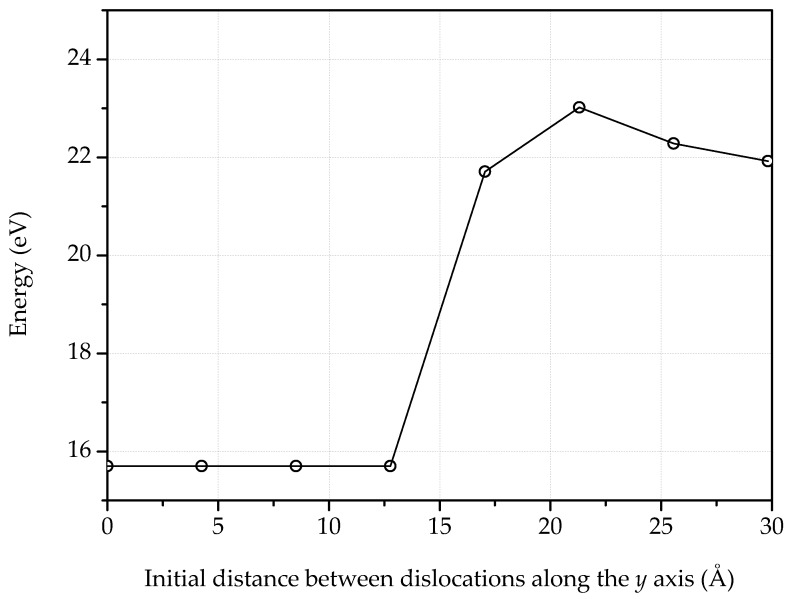
Energy of two n=9 dipoles interacting through different layers in bilayer graphene, as a function of the initial distance between them.

Figure 15 shows an additional example of a bilayer with a n=9 dipole in each monolayer displaced 15 Å relative to each other. The energy of the defects in this configuration is 23.0 eV, whereas the sum of the energies of the monolayer defects is 15.9 eV. The strong steric interference between the deformed monolayers is evident in Figure 15. Figure 16 depicts the energy of two n=9 dipoles, one in each monolayer, as a function of the initial offset distance between dipoles. Remarkably, up to an initial distance of ∼13 Å, the steric interference between the monolayers results in a strong attractive interaction between the dipoles, which relaxed to a zero-distance configuration of energy 15.7 eV. For greater initial distances, the attractive interaction of the dipoles is not strong enough and the dipoles remain offset to each other, resulting in comparatively larger energies.

## 4. Summary and Concluding Remarks

On the basis of calculations carried out on LAMMPS [32] using the LCBOP [30,31] interatomic potential, we have documented the emergence of strong steric effects in bi-layer graphene containing offset dislocations resulting from the out-of-plane displacements of the individual layers.

We have built confidence in the calculations by carefully evaluating the fidelity of the LCBOP potential in a number of configurations. In the harmonic range, we find that the LCBOP potential matches closely the phonon dispersion curves experimentally measured by Siebentritt et al. [33], Oshima et al. [34], Nicklow et al. [35] and Yanagisawa et al. [36]. The predicted phonon dispersion curves are also in good agreement with those predicted by the AIREBO potential [40]. We have also tested the LCBOP potential for dislocation dipole and quadrupole configurations in monolayer graphene. We find that the LCBOP potential is considerably softer, and allows from more complete core relaxation, than other interatomic potentials.

With the LCBOP potential thus validated, we have turned to the characterization of the core structure and energies of dislocation quadrupoles in bilayer graphene. Remarkably, we find that, if the dislocations in the individual layers are sufficiently offset, the out-of-plane displacements of the layers give rise to strong steric interactions which result in high energies. By contrast, if the dislocations are in registry the steric interference between the individual layers is minimized and low energies are attained. We find that there is a critical offset distance between defects in the individual layers that separates both regimes. If the offset between individual-layer defects is less than a critical value, the defects migrate and come into registry in order to minimize their energy. By contrast, if the offset distance is sufficiently large the offset configuration is stable and the steric interaction energy is not relaxed.

Fully coupled thermomechanical-electronic structure calculations of defects in monolayer graphene, including grain boundaries, have been presented in previous work [19]. As noted by numerous authors, the presence of lattice defects profoundly influences the electronic transport properties of graphene, including its band gap structure. However, mechanical free-energy minimization, an effect that has often been neglected in previous analysis, is determinant of—and limits—the geometry and structure of the defects that can arise and be sustained by lattices. This points to the need to account for models of thermomechanical relaxation, of the type presented in this paper, in electronic-structure calculations. Evidently, isolated defects do not exhaust the broad range of lattice defect structures that do occur stably in bilayer graphene. For instance, antiphase boundaries are found to separate AB and BA domains [10,49] and intricate atomic and electronic reconstructions are found to arise in twisted bilayer graphene [50,51], among other structures. These and other similar phenomena suggest they could be worthwhile directions for further study. 

## Figures and Tables

**Table 1:** Parameters of the force constants models [N/m] by Wirtz et al. [25], Tewary et al. [27], Ariza et al. [38,40], Mendez et al. [18] and the present work.

	[25]	[27]	[38]	[40]	[18]	Present Work
$\alpha_{1}$	399.0	409.7	364.0	527.7	497.2	423.6
$\beta_{1}$	135.7	145.0	247.0	68.1	173.7	144.3
$\delta_{1}$	292.8	98.9	100.5	118.3	106.9	75.7
$\alpha_{2}$	−79.6	−40.8	−30.8	5.8	−41.43	−6.5
$\beta_{2}$	67.8	74.2	72.3	32.7	58.1	29.9
$\gamma_{2}$	39.2	−9.1	−17.8	26.7	−3.0	−23.7
$\delta_{2}$	0.9	−8.2	−11.5	−16.9	−15.9	−8.8
$\alpha_{3}$	0.0	−33.2		0.0	−20.64	4.0
$\beta_{3}$	0.0	50.1		0.0	34.51	−0.8
$\delta_{3}$	−34.3	5.8		3.7	9.1	−0.8
$\alpha_{4}$	0.0	10.5		0.0		0.3
$\beta_{4}$	0.0	5.0		0.0		0.0
$\gamma_{4}$	0.0	2.2		0.0		0.1
$\tau_{4}$	0.0	−2.2		0.0		0.1
$\delta_{4}$	17.1	−5.2		−1.8		0.0
